# Protective Effect of *Lactobacillus rhamnosus* GG on TiO_2_ Nanoparticles-Induced Oxidative Stress Damage in the Liver of Young Rats

**DOI:** 10.3390/nano11030803

**Published:** 2021-03-21

**Authors:** Penghui Nie, Mengqi Wang, Yu Zhao, Shanji Liu, Ling Chen, Hengyi Xu

**Affiliations:** State Key Laboratory of Food Science and Technology, Nanchang University, Nanchang 330047, China; 6300817509@email.ncu.edu.cn (P.N.); 402313320073@email.ncu.edu.cn (M.W.); 5603515014@email.ncu.edu.cn (Y.Z.); 412314919032@email.ncu.edu.cn (S.L.); 401441518162@email.ncu.edu.cn (L.C.)

**Keywords:** titanium dioxide nanoparticles, *Lactobacillus rhamnosus* GG, liver, toxicity, oxidative stress

## Abstract

The potential toxicity of titanium dioxide nanoparticles (TiO_2_ NPs) to mammals has become a widespread concern. Young individuals exposed to TiO_2_ NPs have a higher risk than adults. In this study, the protective effects of *Lactobacillus rhamnosus* GG (LGG) on liver toxicity in young rats induced by TiO_2_ NPs were explored. Results show that the four-week-old rats that underwent LGG after the oral intake of TiO_2_ NPs could prevent weight loss, reduce hematological indicators (WBC and NEUT) and serum biochemical indicators (AST, ALT, AST/ALT, and ALP). Moreover, it alleviated the pathological damage of the liver (as indicated by the disordered hepatocytes, more eosinophilic, ballooning degeneration, and accompany with blood cells), but it did not reduce the Ti contents in the liver. In addition, RT-qPCR results indicated that LGG restored the expression of anti-oxidative stress-related genes, such as *SOD1*, *SOD2*, *CAT*, *HO-1*, *GSH*, *GCLC*, and *GCLM* in the liver. In summary, the hepatotoxicity of TiO_2_ NPs in young rats is closely related to oxidative stress, and the antioxidant effect of LGG might protect the harmful effects caused by TiO_2_ NPs.

## 1. Introduction

Recently, nanomaterials have been widely used in various fields such as the chemical industry, food industry, cosmetics, and textiles [1]. In addition, nanomaterials play an important role in clinical and experimental medicine [2,3,4], which made huge impacts on our daily life. Titanium dioxide nanoparticles (TiO_2_ NPs) possess unique physical and chemical properties and have been extensively used in various fields, such as paint, printing ink, rubber, paper, cosmetics, sunscreen, medicine, food additives, and automotive materials [5,6,7,8,9]. Thus, human exposure is likely during the handing and use of freely dispersed TiO_2_ NPs [10], causing concerns about its possible health effects. An increasing number of researches have confirmed the harm of TiO_2_ NPs. In vivo studies have presented that TiO_2_ NPs can induce lung injury, brain injury, liver toxicity, nephrotoxicity, embryotoxicity, and neurotoxicity [11,12,13,14,15].

Notably, young individuals exposed to TiO_2_ NPs are more sensitive than adults [16,17,18]. In addition, American adults are currently exposed to about 0.2–0.7 mg Ti/kg/day, while a child potentially consumes 2–4 times as much Ti as an adult amounting to 1–2 mg Ti/kg/day. Similarly, the UK population is exposed to 2–3 mg Ti/kg/day for children and approximately 1 mg Ti/kg/day for adults [19]. Hence, children are more exposed to TiO_2_ NPs than adults. Research on the toxicity of TiO_2_ NP to young people are lacking. Therefore, relevant studies are urgently needed.

The mechanisms of the toxicity of these NPs should be determined. The oxidative stress (OS) induced by NPs has become one of the toxic mechanisms of NPs [20,21]. OS refers to the excessive production of highly active molecules in the body, including active oxygen free radicals when responding to various harmful stimuli, and the level of oxidation exceeds the antioxidant capacity of the cell to remove oxides. The oxidation system and the antioxidant system are out of balance, resulting in tissue damage [22,23]. Probiotics have antioxidant activity and can reduce damage caused by OS. The supernatant extract of cultured *Bifidobacteria* can scavenge hydroxyl free radicals and superoxide anions and enhance the antioxidant enzyme activity of mice [24]. The high-fat diet fed with *Lactobacillus plantarum* P-8 to the mice raised the antioxidant capacity, thus reducing liver fat accumulation while protecting liver function [25]. *Lactobacillus rhamnosus* GG (LGG) *is* a widely studied probiotic [26], and the previous research of our group confirmed that LGG has a certain repair effect on intestinal injury [27], also it possesses strong antioxidant capacity [28,29].

The liver is an organ with major metabolic function in the body and plays an important part in the metabolism and biotransformation of toxic substances [30,31]. Thus, regardless of the type of injury or functional impairment, hepatotoxicity occurs, leading to health complications. Therefore, the liver is also particularly vulnerable to TiO_2_ NPs.

Accordingly, the protective effect of LGG on the liver toxicity caused by TiO_2_ NPs in young rats was explored in this work. The physiological status of liver effects of TiO_2_ NPs with and without the LGG was assessed by analyzing the hematology, serum biochemistry, and Ti contents in the liver plus the changes in liver morphology. The mechanism of the toxicity effects of TiO_2_ NPs was further explored from the molecular by detecting the gene expression related to OS by using RT-qPCR assay.

## 2. Materials and Methods

### 2.1. Preparation and Characterization of TiO_2_ NPs

TiO_2_ NPs were obtained from Aladdin Industrial Corporation (Shanghai, China). The size and morphology of this material were evaluated via a scanning electron microscope (SEM), and the hydrodynamic diameter in water was evaluated via dynamic light scattering (DLS). Before the experiment, TiO_2_ NPs were weighed and mixed with 1% phosphate buffer saline (PBS). The mixture was ultrasonically treated for 30 min and then vortexed for 5 min by an analog vortex mixer to ensure that TiO_2_ NPs were evenly dispersed in an aqueous solution.

### 2.2. Probiotics Preparation

LGG was cultured in sterile de Man Rogosa Sharpe broth (MRS, Solarbio Science and Technology Co. Ltd., Beijing, China) in an anaerobic circumstance at 37 °C, for 16 h. Then the compound of LGG and MRS broth was centrifuged at 12,000 rpm for 2 min to remove the supernatant of the broth, the pellet washed, and then resuspended in 1% PBS. The LGG was adjusted to 10^8^ CFU/mL 200 μL.

### 2.3. Animals Administration

Female Sprague Dawley (SD) rats (4-week-old, 60 ± 5 g) were obtained from the Jiangxi University Experimental Animal Center of Traditional Chinese Medicine. All animal procedures in this work follow the requirements of the Institutional Animal Care Committee guidelines and have been allowed by the Animal Care Review Committee (approval number 0064257) of Nanchang University, Jiangxi province, China. The female rats were provided adequate food and distilled water, kept in plastic cages in animal rooms at 22 ± 1 °C and relative humidity was 60 ± 10% for. The rats were sorted into 4 groups (n = 6), randomly: the control group (treated with 1% PBS), TiO_2_ NPs (150 mg/kg) group, TiO_2_ NPs (150 mg/kg) + LGG (10^8^ Colony-Forming Units/mL (CFU/mL)) group, and LGG (10^8^ CFU/mL) group, rats in groups TiO_2_ NPs and TiO_2_ NPs + LGG were orally gavaged with TiO_2_ NPs, two hours later, rats of groups TiO_2_ NPs + LGG and LGG were gavaged with LGG (dissolved in 1% PBS). The dosage of TiO_2_ NPs is according to the study of Wang Y et al. [17]. The four groups of rats were given intragastrically for 7 days, rats were weighed and body weight was recorded daily. On the first day after the end of gavage, the rats were weighed and euthanized, the blood samples were obtained through eyeball extraction, the organs and tissues were collected, weighed, next kept at −80 °C for further analysis.

### 2.4. Organ Coefficient

The collected organs including heart, liver, spleen, lung, kidney, brain, thymus, ovary, and uterus were washed with 4 °C saline, dried with filter paper, weighed, and organ coefficients measured.

### 2.5. Analysis of Hematology

The collected blood for hematology analysis. The indicators were detected include white blood cells (WBC), lymphocytes (Lymph), monocytes (Mon), neutrophils (NEUT), red blood cells (RBC), hemoglobin (HGB), and platelets (PLT), which were determined by Adicon clinical laboratories (Nanchang, Jiangxi, China).

### 2.6. Evaluation of Serum Biochemistry

The blood collected in the centrifuge tube was centrifuged at 1000 rpm for 10 min at 4 °C to take the supernatant, and the liver function-related indicators including aspartate aminotransferase (AST), alanine aminotransferase (ALT), and alkaline phosphate enzymes (ALP) were measured. These steps were carried out according to the standard procedures of the reagent test kit (Jiancheng Institute of Bioengineering, NanJing).

### 2.7. Analysis Contents of Ti

Approximately 0.05–0.1 g samples of the liver were dissolved with 1 mL of hydrogen nitrate and 200 μL of Neoprene Rubber, respectively. And heated to 280 °C until the digestion solution was nearly dried out after cooling to ambient temperature, and every sample was blended with ultrapure water, leading to a final volume of 5 mL. Inductively coupled plasma-mass spectrometry (ICP-MS, Varian 820-MS, Palo Alto, CA, USA) was used to analyze the element Ti contents.

### 2.8. Histopathological Examination of Liver

The liver was assessed for histopathological changes. The liver was transferred to 4% formalin solution immediately after harvest. Each sample was embedded in paraffin, sliced into 5 μm thick sections, next put on the slide and stained with hematoxylin and eosin (HE). Histological images were acquired by a Nikon Ti optical microscope (Tokyo, Japan).

### 2.9. Analysis of Gene Expression

According to the manufacturer’s protocol, the AxyPrep Multisource Total RNA Miniprep Kit (Axygen Scientific, CA, US) was used to extract the total RNA of the liver from each group, which was reverse transcribed of total mRNA (1 μg) into cDNA through a Takara PrimeScript TM RT reagent kit (Cat#RR047A, Lot#AK2802). Then the real-time quantitative polymerase chain reaction (qPCR) with TB Green™ Premix Ex Taq™ II (TIi RNaseH Plus, TAKARA Cat#RR820A) was carried out on the CFX Connect™ Real-Time PCR Detection System (Bio-Rad Laboratories, Inc. Louisville, KY, USA). The sequences of primers as presented in Table 1 with glyceraldehyde-3-phosphate dehydrogenase *(GAPDH)* as the internal reference gene. The results were calculated via the 2^−ΔΔCt^ method.

### 2.10. Statistical Analysis

Statistical analyses were executed by SPSS 22.0 software (SPSS, Inc., Chicago, IL, USA) in this work. All the data presented were the means ± SD. One-way analysis of variance (ANOVA) was used to analyze the differences among multiple groups. For all tests, the differences from the control group are represented by * means *p* < 0.05 and ** means *p* < 0.01; the differences from the TiO_2_ NPs group are represented by ^#^ means *p* < 0.05 and ^##^ means *p* < 0.01.

## 3. Results

### 3.1. Characterization of TiO_2_ NPs

**Figure 1:**
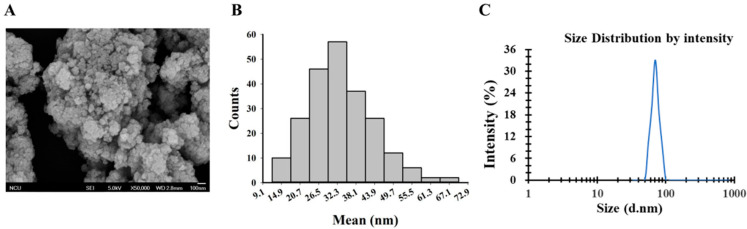
Characterization of titanium dioxide nanoparticles (TiO_2_ NPs). (**A**) TiO_2_ NPs have a spherical geometry as determined by SEM. (**B**) Particles diameter distribution at approximately 33 nm. (**C**) The approximate hydrodynamic size of the particles was 71 nm as determined by DLS analysis.

The observations under SEM indicated that TiO_2_ NPs had a spherical geometry with an average diameter of 33 nm (Figure 1A,B). Based on DLS analysis, the hydrodynamic size of TiO_2_ NPs in water was approximately 71 nm (Figure 1C).

### 3.2. Body Weight Changes in Young Rats

**Figure 2:**
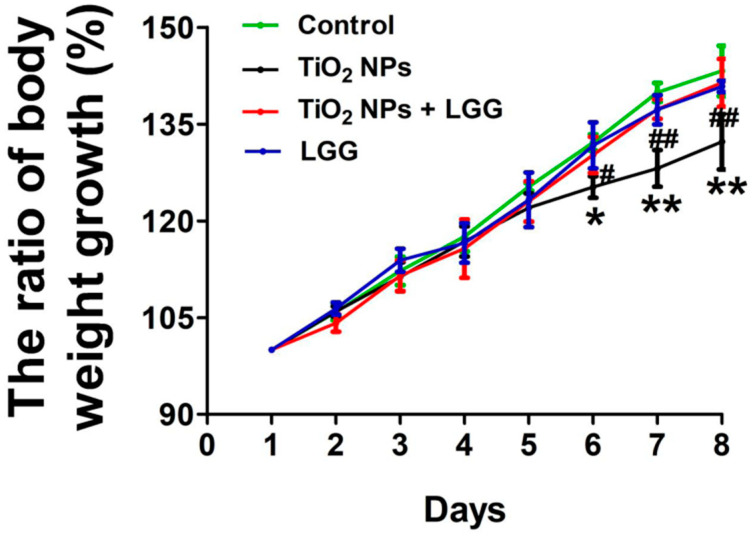
The ratio of body weight growth in the young rats after exposure TiO_2_ NPs. * *p* < 0.05 and ** *p* < 0.01 versus the control group; ^#^ *p* < 0.05 and ^##^ *p* < 0.01 versus the TiO_2_ NPs group. *n* = 6, values are presented as mean ± SD.

The changes in body weight were calculated as shown in Figure 2. The ratio of body weight growth was decreased from the 6th day in the TiO_2_ NPs group than the control group (*p* < 0.05 or *p* < 0.01). Meanwhile, the ratio of body weight growth was increased in the TiO_2_ NPs + LGG group than the TiO_2_ NPs group from the 6th day (*p* < 0.05 or *p* < 0.01).

### 3.3. Organ Coefficient

**Figure 3:**
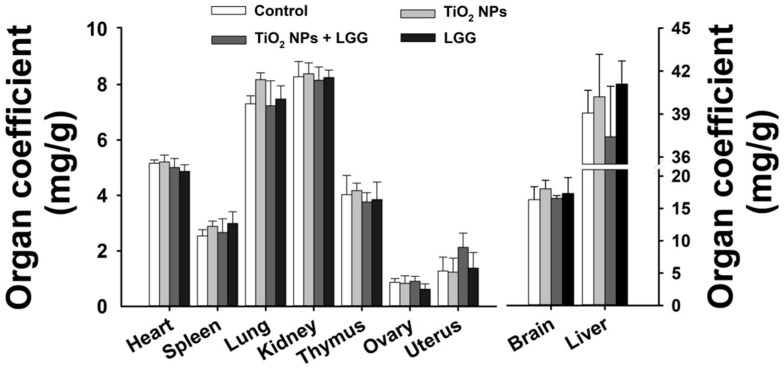
Organ coefficient in the young rats after exposure TiO_2_ NPs. *n* = 6, values are presented as mean ± SD.

Based on the results of the organ coefficients presented in Figure 3, no significant changes were observed in each organ among all the experimental groups.

### 3.4. Hematology

As shown in Table 2, the WBC was 5.57 ± 2.39 × 10^9^ /L and the NEUT was 0.55 ± 0.15 × 10^9^/L in the control group; the WBC was 9.7 ± 1.5 × 10^9^/L and the NEUT was 1.07 ± 0.68 × 10^9^/L in TiO_2_ NPs group, the latter values were higher than the former (*p* < 0.05). The WBC was 6.17 ± 1.05 × 10^9^/L and the NEUT was 0.6 ± 0.1 × 10^9^/L in the TiO_2_ NPs + LGG group, and these values were less than those in the TiO_2_ NP group (*p* < 0.05).

### 3.5. Serum Biochemistry

**Figure 4:**
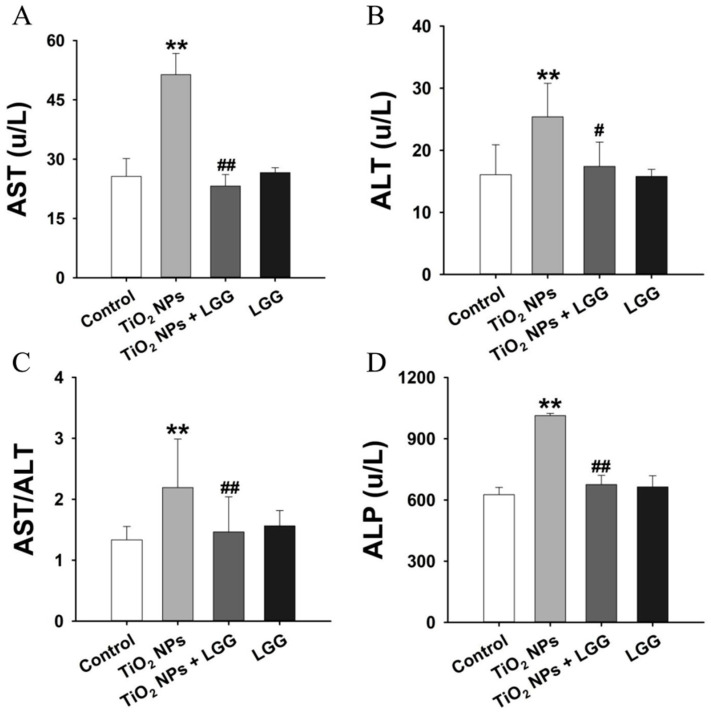
Serum biochemical analysis after oral administration of TiO_2_ NPs in the young rats. (**A**) aspartate aminotransferase, AST. (**B**) alanine aminotransferase ALT. (**C**) The ratio of AST to ALT. (**D**) alkaline phosphatase, ALP. ** *p* < 0.01 versus the control group; ^#^ *p* < 0.05 and ^##^ *p* < 0.01 versus the TiO_2_ NPs group. *n* = 3, values are presented as mean ± SD.

Significantly higher AST, ALT, AST/ALT, and ALP levels were found in the TiO_2_ NPs group than the control. Meanwhile, the AST, ALT, AST/ALT, and ALP levels in the TiO_2_ NPs + LGG group exhibited an obvious decrease compared to the TiO_2_ NPs group (*p* < 0.05) (Figure 4).

### 3.6. Ti Contents in the Liver

**Figure 5:**
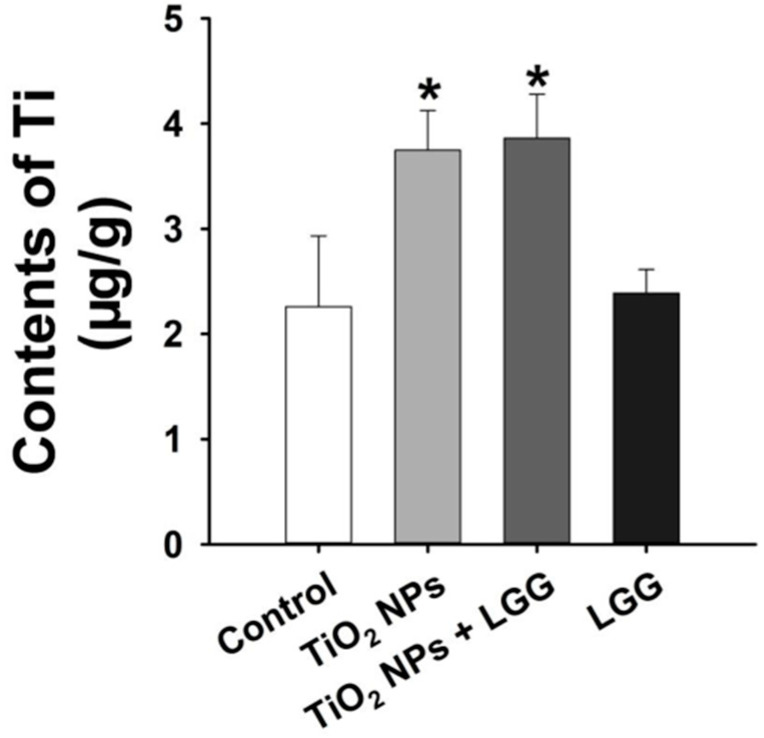
Content of Ti in the liver of young rats after oral exposure to TiO_2_ NPs. * *p* < 0.05 versus the control group. *n* = 3, values are expressed as mean ± SD.

In comparison with the control group, rats exposed to TiO_2_ NPs, with or without LGG, exhibited higher Ti contents in the liver (*p* < 0.05) (Figure 5).

### 3.7. Histopathological Evaluation

**Figure 6:**
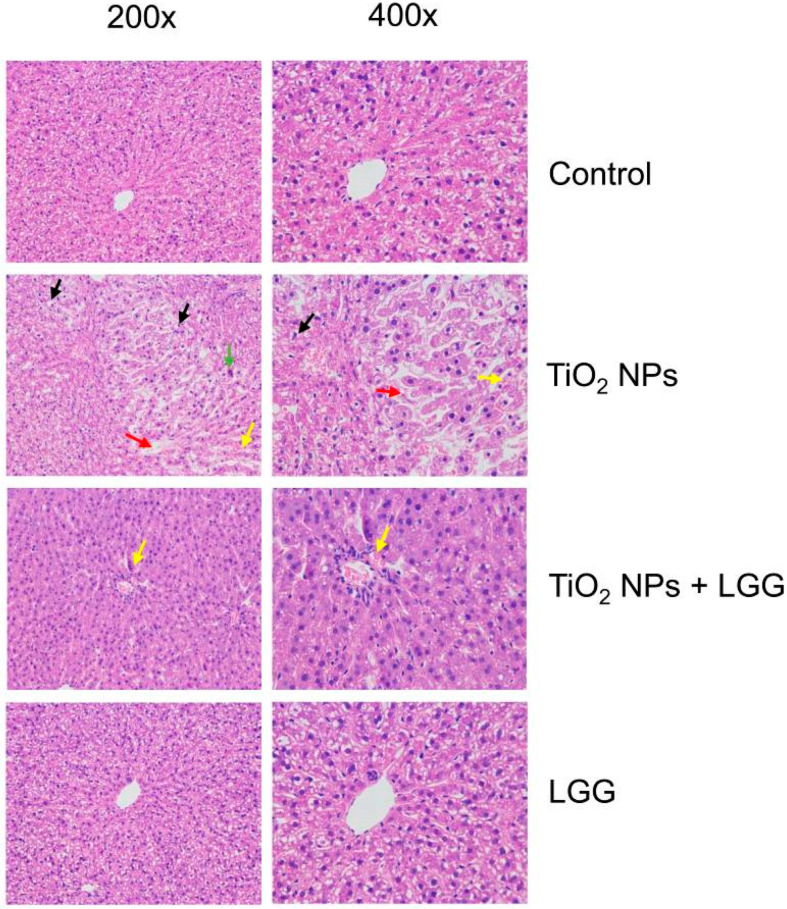
Sections of the liver with HE stained at 200× and 400× of young rats after oral exposure to TiO2 NPs. (black arrows: hepatocytes ballooning degeneration; blue arrows: eosinophilic; red arrows: hepatocytes appear arrangement disordered; yellow arrows: blood cells).

The results of the histopathological evaluation in the liver of rats are presented in Figure 6. After TiO_2_ NPs treatment, hepatocytes appeared in a disordered arrangement, accompanied by many eosinophils, ballooning degeneration, and blood cells. In the TiO_2_ NPs + LGG group, hepatocytes were tightly arranged and accompanied by blood cells.

### 3.8. Levels of Gene Expression

To assess the effect of liver injury in young rats exposed to TiO_2_ NPs, we set the genes of OS expression levels, including *SOD1*, *SOD2*, *HO-1*, *GSH, CAT*, *GCLC*, and *GCLM* for RT-qPCR analysis. The results are shown below.

**Figure 7:**
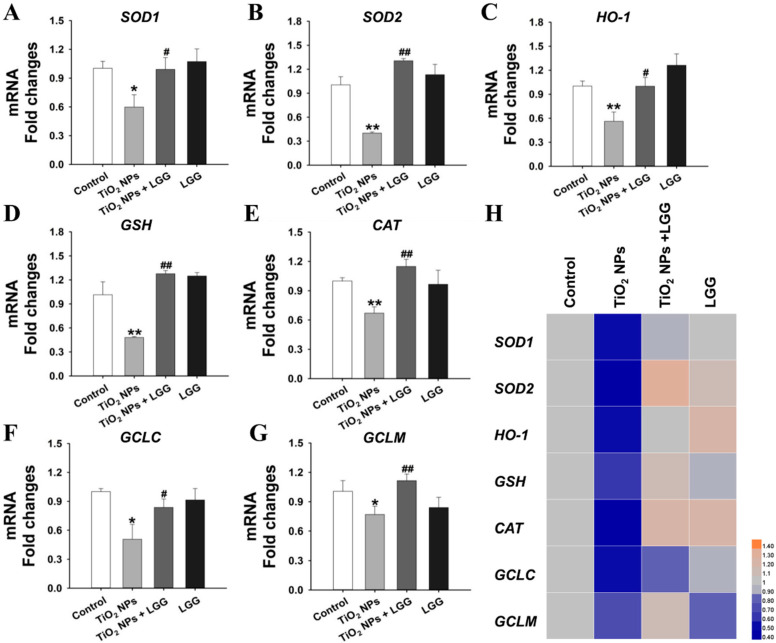
Expression of genes related to OS in the liver of young rats after oral exposure to TiO_2_ NPs. (**A**) *SOD-1*. (**B**) *SOD-2*. (**C**) *HO-1*. (**D**) *GSH*. (**E**) *CAT*. (**F**) *GCLC*. (**G**) *GCLM*. (**H**) Gene thermogram: blue represents low expression and yellow represents high expression, * *p* < 0.05 and ** *p* < 0.01 versus the control group; ^#^ *p* < 0.05 and ^##^ *p* < 0.01 versus the TiO_2_ NPs group. *n* = 3, values are expressed as mean ± SD.

The levels of *SOD1*, *SOD2*, *HO-1*, *GSH, CAT*, *GCLC*, and *GCLM* were obviously downregulated in the TiO_2_ NPs group than the control (*p* < 0.05 or *p* < 0.01). After the young rats exposed to TiO_2_ NPs were treated with LGG, the above genes were significantly increased compared to the TiO_2_ NPs group (*p* < 0.05 or *p* < 0.01) (Figure 7).

## 4. Discussion

TiO_2_ NPs have a wide range of applications and are prone to human exposure. In comparison with adults, children are more exposed to NPs. The oral administration of TiO_2_ NPs in young rats is more toxic than in adult rats [17]. Therefore, in this study, the effect of TiO_2_ NPs on the hepatotoxicity of four-week-old rats was explored. In addition, the protective effect of LGG on the hepatotoxicity of TiO_2_ NPs in young rats and its possible toxicity mechanism were clarified.

The general and physiological conditions including the body weight, organs coefficients, and hematological indexes were evaluated. After the young rats were orally administrated to TiO_2_ NPs for 7 days, which showed that the ratio of body weight growth was significantly decreased (Figure 2) and the organ coefficients had no significant changes (Figure 3). The hematological analysis presented that the levels of WBC and NEUT were significantly increased (Table 2). Similar studies have found that acute administration to TiO_2_ NPs can cause inflammation in mice [32]. Serum biochemical indicators of liver damage [33,34,35] results showed that the TiO_2_ NPs treatment remarkably increased the level of AST, ALT, ALP, and the AST/ALT ratio (Figure 4), indicating the liver damage induced by TiO_2_ NPs. LGG treatment of rats exposed to TiO_2_ NPs obviously reduced the parameters of liver function (AST, ALT, and ALP), indicating that LGG has a protective effect on liver function [36,37].

Hepatocyte degeneration and other pathological changes also verified that TiO_2_ NPs lead to liver damage (Figure 6), thus supporting that TiO_2_ NPs lead to pathological changes in the liver. Our research results are similar to previous researches [38,39]. This phenomenon might be caused by the damage leading to the accumulation of NPs in the liver (Figure 5). A similar study reported the distribution of TiO_2_ NPs in mouse tissues and organs, causing damage [40]. After LGG treatment, the liver damage of young rats was relieved, but the Ti contents in the liver did not significantly decrease (Figure 5), which indicated that LGG did not reduce tissue damage by inhibiting Ti content, while it might enhance the body’s resistance to TiO_2_ NPs. LGG can be used as a stable protectant to prevent body damage [41]. 

To further clarify the effect of LGG on the liver of young rats exposed to TiO_2_ NPs, we explored the TiO_2_ NPs toxicity mechanism. OS caused by TiO_2_ NPs is the main cause of tissue damage [42,43]. Therefore, OS may cause liver toxicity induced by TiO_2_ NPs. OS is a normal cellular process that involves many aspects of cell signal transduction, while excessive OS may be harmful and cause the degree of oxidation of cells to exceed their antioxidant capacity [44]. Superoxide dismutase (*SOD*) is an important antioxidant enzyme that can eliminate superoxide anion free radicals, the intermediate product of aerobic metabolism in organisms, and is the first line of defense against oxygen free radical damage [45]. The production of oxidative free radicals in the human body reduces the activity of SOD, causing peroxidative damage to membrane lipids, generating a large amount of malondialdehyde (*MDA*) to further damaging cells. Heme oxygenase-1 (*HO-1*) decomposes heme into free iron, biliverdin, and nitric oxide and is considered an antioxidant [46], which plays a significant protective role in OS injury [47]. As an important antioxidant enzyme that exists in almost all biological tissues that utilize oxygen, catalase (*CAT*) uses iron or manganese as a cofactor to catalyze the reduction or degradation of hydrogen peroxide to molecular oxygen and water, thereby accomplishing the detoxification process simulated via *SOD* [48]. Glutathione (*GSH*) is the major cellular defense against ROS for it can remove both hydroxyl radicals and singlet oxygen and limit the levels of certain reactive aldehydes and peroxides within the cell through glutathione transferases and glutathione peroxidases [49,50]. The catalytic (*GCLC)* and regulatory subunits (*GCLM*) are two subunits of glutamate-cysteine ligase (GCL), which is the first rate-limiting enzyme in *GSH* synthesis [51,52,53]. Mice knockout in the *GCLM* or *GCLC* gene significantly reduced GSH content in the liver [54,55]. In this study, as presented in Figure 6, the expression of *SOD-1*, *SOD-2*, *HO-1*, *CAT*, *GSH*, *GCLC*, and *GCLM* in the TiO_2_ NPs treatment group obviously decreased, suggesting that administration of TiO_2_ NPs in young rats caused the activation of the antioxidant system, reflecting the occurrence of OS that caused liver damage.

However, after oral administration of TiO_2_ NPs with LGG in young rats, the expression of the above genes was restored, indicating that LGG can resist OS. This phenomenon occurred because the antioxidant capacity of LGG can significantly alleviate the effect on oxidative damage induced by various stressors. Goyal et al. found that LGG possesses antioxidative properties in Giardia-mediated tissue injury [56]. Sun et al. found that feeding LGG can clear the activity of mice under stress, inhibit the microorganisms that produce reactive oxygen species, and enhance the antioxidant capacity on the body [57]. Therefore, LGG could significantly improve liver damage by inhibiting OS caused by TiO_2_ NPs.

## 5. Conclusions

The protective effects of LGG on liver toxicity in young rats induced by TiO_2_ NPs were explored in this work. Treatment of the rats with LGG after oral administration to TiO_2_ NPs suggests that LGG could prevent the general and physiological toxicity, and alleviate the pathological damage of the liver in young rats. The results of the molecular level studies suggest that the mRNA expression of anti-oxidative stress genes (*SOD1*, *SOD2*, *CAT*, *HO-1*, *GSH*, *GCLC,* and *GCLM*) significantly increased. Hence, TiO_2_ NPs could induce liver damage in young rats through oxidative stress. The antioxidant effect of LGG could have a certain therapeutic effect in preventing and relieving liver damage caused by TiO_2_ NPs. However, the exact mechanism by which LGG participates in antioxidant stress remains uncertain, and further research is needed to completely define the exact antioxidant mechanism of this probiotic in order to achieve antioxidant effects.

## Figures and Tables

**Table 1:** Genes and primers selected for RT-qPCR.

Gene	Primer	Sequence (5′-3′)
*SOD1*	Forward	TTTTGCTCTCCCAGGCCG
	Reverse	ACCGCCATGTTTCTTAGAGTG
*SOD2*	Forward	ACTTGAAACGTGTAACTAGGC
	Reverse	CTTTCATACAATACACAGTCGG
*HO-1*	Forward	TTTTCACCTTCCCAGCAT
	Reverse	TTAGCCTCTTCTGTCACCCT
*CAT*	Forward	ATAGCCAGAAGAGAAACCCACA
	Reverse	CCTCTCCATTCGCATTAACCAG
*GSH*	Forward	ATCCCACTGCGCTCATGACC
	Reverse	AGCCAGCCATCACCAAGCC
*GCLC*	Forward	GAGCGAGATGCCGTCTTACA
	Reverse	TTGCTACACCCATCCACCAC
*GCLM*	Forward	TGTTTGACCAAGTGCCCAT
	Reverse	ATCTAAAATGCCTTCGGTGT
*GAPDH*	Forward	TCCCTCAAGATTGTCAGCAA
	Reverse	AGATCCACAACGGATACATT

**Table 2:** Effects of oral exposure of TiO_2_ NPs on blood routine indexes in the young rats.

Indexes	Control	TiO_2_ NPs	TiO_2_ NPs + LGG	LGG
WBC (10^9^/L)	5.57 ± 2.39	9.7 ± 1.5 *	6.17 ± 1.05 ^#^	6.43 ± 2.01
Lymph (10^9^/L)	4.8 ± 2.11	6.17 ± 2.67	5.3 ± 1.19	4.85 ± 1.75
Mon (10^9^/L)	0.1 ± 0.08	0.18 ± 0.15	0.12 ± 0.02	0.13 ± 0.05
NEUT (10^9^/L)	0.55 ± 0.15	1.07 ± 0.68 *	0.6 ± 0.1 ^#^	0.7 ± 0.22
RBC (10^12^/L)	2.33 ± 1.08	2.44 ± 0.77	2.12 ± 0.26	2.26 ± 0.15
HGB (g/L)	85.67 ± 22.51	94.5 ± 17.64	81 ± 15.9	92 ± 13.93
PLT (10^12^/L)	930.33 ± 340.19	687.67 ± 121.5	1072.5 ± 25.5	896.5 ± 85.5

* *p* < 0.05 versus the control group; ^#^ *p* < 0.05 versus the TiO_2_ NPs group. *n* = 3, values are presented as mean ± SD.

## Data Availability

The data used to support the findings of this study are included within the article.

## Abbreviations

TiO_2_ NPs: Titanium dioxide nanoparticles; LGG: *Lactobacillus rhamnosus* GG; OS: oxidative stress; SEM: scanning electron microscope; DLS: dynamic light scattering; PBS: phosphate buffer saline; CFU: Colony-Forming Units; SD: Sprague Dawley; WBC: white blood cells; Lymph: lymphocytes; Mon: monocytes; NEUT: neutrophils; RBC: red blood cells; HGB: hemoglobin; PLT: platelets; AST: aspartate aminotransferase; ALT: alanine aminotransferase; ALP: alkaline phosphate enzymes; ICP-MS: Inductively coupled plasma-mass spectrometry; HE: hematoxylin-eosin; RT-qPCR: real-time quantitative polymerase chain reaction; GAPDH: glyceraldehyde-3-phosphate dehydrogenase; ANOVA: One-way analysis of variance; SOD: superoxide dismutase; HO-1: heme oxygenase-1; CAT: catalase; GSH: glutathione; GCLC: catalytic of glutamate cysteine ligase; GCLM: modifier of glutamate cysteine ligase.

## References

[1] Peters R.J., Bouwmeester H., Gottardo S., Amenta V., Arena M., Brandhoff P., Marvin H.J., Mech A., Moniz F.B., Pesudo L.Q. (2016). Nanomaterials for products and application in agriculture, feed and food. Trends Food Sci. Technol..

[2] Das S.S., Bharadwaj P., Bilal M., Barani M., Rahdar A., Taboada P., Bungau S., Kyzas G.Z. (2020). Stimuli-Responsive Polymeric Nanocarriers for Drug Delivery, Imaging, and Theragnosis. Polymer.

[3] Chandra H., Singh C., Kumari P., Yadav S., Mishra A.P., Laishevtcev A., Brisc C., Brisc M.C., Munteanu M.A., Bungau S. (2020). Promising Roles of Alternative Medicine and Plant-Based Nanotechnology as Remedies for Urinary Tract Infections. Molecules.

[4] Santos B.T., Pérez C.F., Bourzac J.F.I., Nápoles Y.M., González W.R., Bourg V., Torralba D., Pérez V., Mouriño A., Ayala J. (2019). Remote induction of cellular immune response in mice by anti-meningococcal nanocochleates-nanoproteoliposomes. Sci. Total Environ..

[5] Shen S., Chen J., Wang M., Sheng X., Chen X., Feng X., Mao S.S. (2018). Titanium dioxide nanostructures for photoelectrochemical applications. Prog. Mater. Sci..

[6] Dréno B., Alexis A., Chuberre B., Marinovich M. (2019). Safety of titanium dioxide nanoparticles in cosmetics. J. Eur. Acad. Dermatol. Venereol..

[7] Nasr M., Eid C., Habchi R., Miele P., Bechelany M. (2018). Recent Progress on Titanium Dioxide Nanomaterials for Photocatalytic Applications. ChemSusChem.

[8] Yemmireddy V.K., Hung Y.C. (2015). Selection of photocatalytic bactericidal titanium dioxide (TiO2) nanopar-ticles for food safety applications. LWT Food Sci. Technol..

[9] Ziental D., Czarczynska-Goslinska B., Mlynarczyk D.T., Glowacka-Sobotta A., Stanisz B., Goslinski T., Sobotta L. (2020). Titanium Dioxide Nanoparticles: Prospects and Applications in Medicine. Nanomaterials.

[10] Morimoto Y., Izumi H., Yoshiura Y., Tomonaga T., Lee B.W., Okada T., Oyabu T., Myojo T., Kawai K., Yatera K. (2016). Comparison of pulmonary inflammatory responses following intratracheal instillation and inhalation of nanopar-ticles. Nanotoxicology.

[11] Zhao L., Zhu Y., Chen Z., Xu H., Zhou J., Tang S., Xu Z., Kong F., Li X., Zhang Y. (2018). Cardiopulmonary effects induced by occupational exposure to titanium dioxide nanoparticles. Nanotoxicology.

[12] Jia X., Wang S., Zhou L., Sun L. (2017). The Potential Liver, Brain, and Embryo Toxicity of Titanium Dioxide Nanoparticles on Mice. Nanoscale Res. Lett..

[13] Gao X., Yin S., Tang M., Chen J., Yang Z., Zhang W., Chen L., Yang B., Li Z., Zha Y. (2011). Effects of Developmental Exposure to TiO_2_ Nanoparticles on Synaptic Plasticity in Hippocampal Dentate Gyrus Area: An In Vivo Study in Anesthetized Rats. Biol. Trace Elem. Res..

[14] Liu R., Zhang X., Pu Y., Yin L., Li Y., Zhang X., Liang G., Li X., Zhang J. (2010). Small-sized titanium dioxide nanoparticles mediate immune toxicity in rat pulmonary alveolar macrophages in vivo. J. Nanosci. Nanotechnol..

[15] Hong J., Zhang Y.-Q. (2016). Murine liver damage caused by exposure to nano-titanium dioxide. Nanotechnol..

[16] Wang Y., Chen Z.-J., Ba T., Pu J., Cui X.-X., Jia G. (2014). Effects of TiO_2_; nanoparticles on antioxidant function and element content of liver and kidney tissues in young and adult rats. Beijing Da Xue Xue Bao Yi Xue Ban = J. Peking Univ. Health Sci..

[17] Wang Y., Chen Z., Ba T., Pu J., Chen T., Song Y., Gu Y., Qian Q., Xu Y., Xiang K. (2013). Susceptibility of Young and Adult Rats to the Oral Toxicity of Titanium Dioxide Nanoparticles. Small.

[18] Hu H., Zhang B., Li L., Guo Q., Yang D., Wei X., Fan X., Liu J., Wu Q., Oh Y. (2019). The toxic effects of titanium dioxide nanoparticles on plasma glucose metabolism are more severe in developing mice than in adult mice. Environ. Toxicol..

[19] Weir A., Westerhoff P., Fabricius L., Hristovski K., Von Goetz N. (2012). Titanium Dioxide Nanoparticles in Food and Personal Care Products. Environ. Sci. Technol..

[20] Assadian E., Dezhampanah H., Seydi E., Pourahmad J. (2019). Toxicity of Fe_2_O_3_ nanoparticles on human blood lymphocytes. J. Biochem. Mol. Toxicol..

[21] Saliani M., Jalal R., Goharshadi E.K. (2016). Mechanism of oxidative stress involved in the toxicity of ZnO nanoparticles against eukaryotic cells. Nanomed. J..

[22] Shukla R.K., Kumar A., Vallabani N.V.S., Pandey A.K., Dhawan A. (2014). Titanium dioxide nanoparticle-induced oxidative stress triggers DNA damage and hepatic injury in mice. Nanomedicine.

[23] Azim S.A.A., Darwish H.A., Rizk M.Z., Ali S.A., Kadry M.O. (2015). Amelioration of titanium dioxide nanoparticles-induced liver injury in mice: Possible role of some antioxi-dants-ScienceDirect. Exp. Toxicol. Pathol..

[24] Shen Q., Shang N., Li P. (2010). In Vitro and In Vivo Antioxidant Activity of Bifidobacterium animalis 01 Isolated from Centenarians. Curr. Microbiol..

[25] Bao Y., Wang Z., Zhang Y., Zhang J., Wang L., Dong X., Su F., Yao G., Wang S., Zhang H. (2012). Effect ofLactobacillus plantarumP-8 on lipid metabolism in hyperlipidemic rat model. Eur. J. Lipid Sci. Technol..

[26] Vandenplas Y., Huys G., Daube G. (2015). Probiotics: An update. J. de Pediatr..

[27] Zhao Y., Tang Y., Chen L., Lv S., Liu S., Nie P., Aguilar Z.P., Xu H. (2020). Restraining the TiO_2_ nanoparticles-induced intestinal inflammation mediated by gut microbiota in juvenile rats via ingestion of Lactobacillus rhamnosus GG. Ecotoxicol. Environ. Saf..

[28] Iglesias M., Echeverría G., Viñas I., López M., Abadias M. (2018). Biopreservation of fresh-cut pear using Lactobacillus rhamnosus GG and effect on quality and volatile compounds. LWT.

[29] Jia Z., Pang X., Lv J. (2018). Reduced-Fat Response of Lactobacillus casei subsp. casei SY13 on a Time and Dose-Dependent Model. Front. Microbiol..

[30] Casotti V., D’Antiga L. (2019). Basic principles of liver physiology. Pediatric Hepatology and Liver Transplantation.

[31] Mossa A.T.H., Refaie A.A., Ramadan A., Bouajila J. (2013). Amelioration of Prallethrin-Induced Oxidative Stress and Hepatotoxicity in Rat by the Administration of Ori-ganum majorana Essential Oil. BioMed Res. Int..

[32] Xu J., Shi H., Ruth M., Yu H., Lazar L., Zou B., Yang C., Wu A., Zhao J. (2013). Acute Toxicity of Intravenously Administered Titanium Dioxide Nanoparticles in Mice. PLoS ONE.

[33] Ashar W.M.P., Muthu M.H.S. (2012). Fenvalerate induced hepatotoxicity and its amelioration by quercetin. Int. J. Pharm. Res..

[34] Esmaeillou M., Moharamnejad M., Hsankhani R., Tehrani A.A., Maadi H. (2013). Toxicity of ZnO nanoparticles in healthy adult mice. Environ. Toxicol. Pharmacol..

[35] Zhang L.-X., Lv Y., Xu A.-M., Wang H.-Z. (2019). The prognostic significance of serum gamma-glutamyltransferase levels and AST/ALT in primary hepatic carcinoma. BMC Cancer.

[36] Gratz S.W., Mykkanen H., El-Nezami H.S. (2010). Probiotics and gut health: A special focus on liver diseases. Word J. Gastroenterol..

[37] Bouhafs L., Moudilou E.N., Exbrayat J.M., Lahouel M., Idoui T. (2015). Protective effects of probioticLactobacillus plantarumBJ0021 on liver and kidney oxidative stress and apoptosis induced by endosulfan in pregnant rats. Ren. Fail..

[38] Hassanein K.M., El-Amir Y.O. (2017). Protective effects of thymoquinone and avenanthramides on titanium dioxide nanoparticles induced toxicity in Sprague-Dawley rats. Pathol. Res. Pract..

[39] Suker D.K., Jasim F.A. (2018). Liver histopathological alteration after repeated intra-tracheal instillation of titanium dioxide in male rats. Gastroenterol. Hepatol. Bed Bench.

[40] Yao L., Chen L., Chen B., Tang Y., Zhao Y., Liu S., Xu H. (2021). Toxic effects of TiO_2_ NPs in the blood-milk barrier of the maternal dams and growth of offspring. Ecotoxicol. Environ. Saf..

[41] Wang Y., Liu Y., Kirpich I., Ma Z., Wang C., Zhang M., Suttles J., McClain C., Feng W. (2013). Lactobacillus rhamnosus GG reduces hepatic TNFα production and inflammation in chronic alcohol-induced liver injury. J. Nutr. Biochem..

[42] Di Zhou S.H., Yan T., Long C., Xu J., Zheng P., Chen Z., Jia G. (2019). Toxicity of titanium dioxide nanoparticles induced by reactive oxygen species. React. Oxyg. Species.

[43] Hu H., Fan X., Yin Y., Guo Q., Yang D., Wei X., Zhang B., Liu J., Wu Q., Oh Y. (2019). Mechanisms of titanium dioxide nanoparticle-induced oxidative stress and modulation of plasma glucose in mice. Environ. Toxicol..

[44] Huang Y.-W., Wu C.-H., Aronstam R.S. (2010). Toxicity of Transition Metal Oxide Nanoparticles: Recent Insights from in vitro Studies. Materials.

[45] Moreau J.-L., Jenck F., Martin J.R., Perrin S., Haefely W.E. (1993). Effects of repeated mild stress and two antidepressant treatments on the behavioral response to 5HT1C receptor activation in rats. Psychopharmacology.

[46] Choi A.M., Alam J. (1996). Heme oxygenase-1: Function, regulation, and implication of a novel stress-inducible protein in oxidant-induced lung injury. Am. J. Respir. Cell Mol. Biol..

[47] Loboda A., Damulewicz M., Pyza E., Jozkowicz A., Dulak J. (2016). Role of Nrf2/HO-1 system in development, oxidative stress response and diseases: An evolutionarily conserved mechanism. Cell. Mol. Life Sci..

[48] Tehrani H.S., Moosavi-Movahedi A.A. (2018). Biology, Catalase and its mysteries. Prog. Biophys. Mol. Biol..

[49] Korge P., Calmettes G., Weiss J.N. (2015). Increased reactive oxygen species production during reductive stress: The roles of mitochondrial glutathione and thioredoxin reductases. Biochim. Biophys. Acta Bioenerg..

[50] Gill S.S., Tuteja N. (2010). Reactive oxygen species and antioxidant machinery in abiotic stress tolerance in crop plants. Plant Physiol. Biochem..

[51] Lu S.C. (2009). Regulation of glutathione synthesis. Mol. Asp. Med..

[52] Franklin C.C., Backos D.S., Mohar I., White C.C., Forman H.J., Kavanagh T.J. (2009). Structure, function, and post-translational regulation of the catalytic and modifier subunits of glutamate cysteine ligase. Mol. Asp. Med..

[53] Ayer A., Zarjou A., Agarwal A., Stocker R. (2016). Heme Oxygenases in Cardiovascular Health and Disease. Physiol. Rev..

[54] Weldy C.S., Luttrell I.P., White C.C., Morgan-Stevenson V., Bammler T.K., Beyer R.P., Afsharinejad Z., Kim F., Chitaley K., Kavanagh T.J. (2012). Glutathione (GSH) and the GSH synthesis gene Gclm modulate vascular reactivity in mice. Free. Radic. Biol. Med..

[55] Chen Y., Yang Y., Miller M.L., Shen D., Shertzer H.G., Stringer K.F., Wang B., Schneider S.N., Nebert D.W., Dalton T.P. (2007). Hepatocyte-specific Gclc deletion leads to rapid onset of steatosis with mitochondrial injury and liver failure. Hepatology.

[56] Goyal N., Rishi P., Shukla G. (2013). Lactobacillus rhamnosus GG antagonizes Giardia intestinalis induced oxidative stress and intestinal disaccharidases: An experimental study. World J. Microbiol. Biotechnol..

[57] Sun J., Hu X.-L., Le G.-W., Shi Y.-H. (2012). Inhibition of Fe-induced colon oxidative stress by lactobacilli in mice. World J. Microbiol. Biotechnol..

